# Asymmetric Organocatalysis at the Service of Medicinal Chemistry

**DOI:** 10.1155/2014/531695

**Published:** 2014-03-11

**Authors:** Alfredo Ricci

**Affiliations:** Department of Industrial Chemistry “Toso Montanari”, School of Science, University of Bologna, V. Risorgimento 4, 40136 Bologna, Italy

## Abstract

The application of the most representative and up-to-date examples of homogeneous asymmetric organocatalysis to the synthesis of molecules of interest in medicinal chemistry is reported. The use of different types of organocatalysts operative via noncovalent and covalent interactions is critically reviewed and the possibility of running some of these reactions on large or industrial scale is described. A comparison between the organo- and metal-catalysed methodologies is offered in several cases, thus highlighting the merits and drawbacks of these two complementary approaches to the obtainment of very popular on market drugs or of related key scaffolds.

## 1. Introduction

Over the past ten years, the field of enantioselective organocatalysis has had a significant impact on chemical synthesis [1, 2]. Currently, asymmetric organocatalysis is recognized [3] as an independent synthetic tool besides asymmetric metallic catalysis and enzymatic catalysis for the synthesis of chiral organic molecules. Multiple advantages compared with the other two catalytic domains are the reasons for the rapid growth and acceptance of organocatalysis. In general, organocatalysts are air- and moisture-stable and, thus, inert-equipments such as vacuum lines or glove boxes are not necessary. They are easy to handle even on large scale and relatively less toxic compared to transition metals. Moreover, frequently the reactions are conducted under mild conditions and high concentrations thus avoiding the use of large amounts of solvents and minimizing waste.

**Figure 1:**
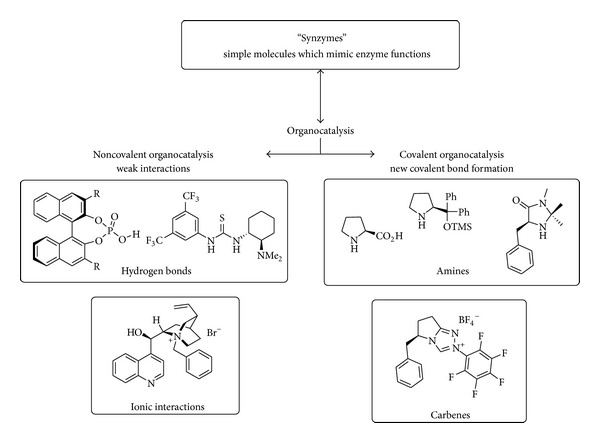
General classification of the activation mode of several representative classes of molecules in organocatalysis.

The organocatalysts can be classified by means of their interactions with the substrate or “mode of action” as covalent or noncovalent catalysts (Figure 1).

In covalent organocatalysis, a new covalent bond between the catalysts and the substrate is formed as in the case of aminocatalysis [4] and carbenes [5], leading to a strong interaction between the substrate and the reagent in the reaction. In the case of noncovalent interactions between the substrate and the catalyst, the activation of the substrate occurs via weak binding exemplified by hydrogen bonding [6] or ionic interaction as in the case of phase transfer catalysis [7].

The field of asymmetric organocatalysis has enjoyed phenomenal growth in the past 15 years [8–10] and during the “golden age” [11] of organocatalysis many researchers from academia and chemical industry were involved in this field, with most efforts focused on the development of novel organocatalysts, new reactivities and asymmetric methodologies. Moreover current developments in the field of the synthesis of structurally complex and/or polyfunctionalized molecules indicate that chemists have adopted the fundamental principles of biosynthesis as synthetic strategic key elements for their synthetic approaches [12]. Among these, cascade reactions [13, 14] employing a single catalyst capable of promoting each single step have gained in the recent years an important role in the efficient and rapid generation of molecules with complex architectures generally correlated with specificity of action and potentially useful biological properties [15]. Organocatalysts turn out to be particularly favourable when used in catalytic cascade reactions because they allow distinct modes of activation, which can often be combined [16, 17].

Despite their great development, the application of organocatalytic methodologies to the synthesis of active compounds in medicinal chemistry in the past years has rarely been reported. However, in most recent years organocatalytic methodologies for the synthesis of enantioenriched molecules for medicinal chemistry purposes have been gaining momentum being particularly attractive for the preparation of compounds that do not tolerate metal contamination. In academia, several groups have made a remarkable effort to show the great applicability of organocatalysts to the total synthesis of bioactive natural products [18] and of drugs [19] most of them currently available in the market such as oseltamivir, warfarin, paroxetine, baclofen, and maraviroc. These efforts mainly focused on the removal of barriers for scale-up by addressing issues such as catalyst loading, product inhibition, substrate scope, and bulk availability of designer catalysts which have drawn the attention of the companies [20, 21] that have begun to incorporate organocatalysis as a synthetic tool in some industrial scale processes [22, 23].

In this review some of the most recent representative applications of asymmetric organocatalysis to medicinal chemistry will be highlighted. Not only the access to market available drugs but also the access to drug candidates, to medicinal scaffolds, and to promising new compounds whose biological profile has not yet been fully explored will be reviewed. In some cases, a comparison between organo- and metal-catalysed methodologies aimed at the obtainment of the same medicinal targets will be reported and a few interesting industrial examples of the use of organocatalysis in medicinal chemistry taken from the literature will be discussed as well.

## 2. Discussion

### 2.1. Noncovalent Organocatalysis

#### 2.1.1. Hydrogen Bonding Catalysis

**Figure 2:**
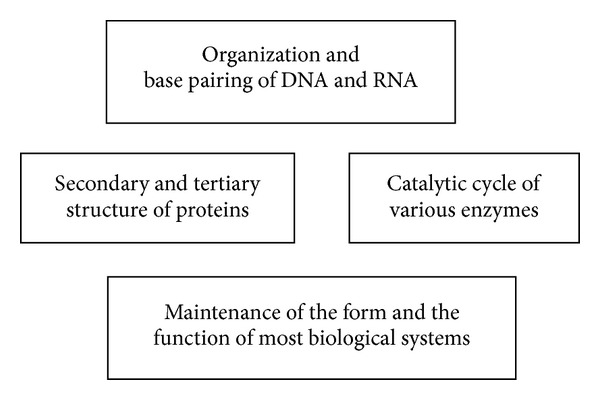
Some of the vital functions that hydrogen bonds fulfil in biological systems.

This ubiquitous interaction is one of the central forces in Nature. As an individual, hydrogen bonds are feeble and quite easy to break. However when acting together they become much stronger and lean each other. This phenomenon is called “cooperativity” (*1* +* 1 is more than 2*). Some of the many vital functions that hydrogen bonds fulfil in biological systems are shown in Figure 2.

The simultaneous donation of two hydrogen bonds leads to a highly successful strategy for electrophilic activation, increased strength, and directionality relative to single hydrogen bonds. Therefore asymmetric catalysis via noncovalent bond interactions such as hydrogen bonding turns out to be a powerful synthetic strategy.

**Figure 3:**
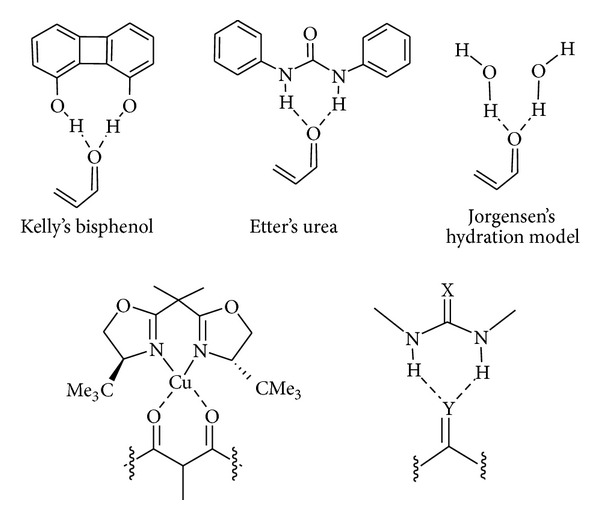
Original achievements regarding bis-hydrogen-bonded complexes.

Original achievements regarding bis-hydrogen bond complexes have been reported through the years. It is worth noting that this two-point binding is a powerful strategy both in metal-centered catalysis and in organocatalysis as shown in Figure 3.

However, whereas coordination to a chiral Lewis acid imposes limitations upon substrate structure, any Lewis base is in principle capable of engaging bifurcated hydrogen bonds so that this catalytic strategy has potential as a general paradigm for synthesis. Thiourea catalysts designed by Sigman and Jacobsen [24] and Corey and Grogan [25] as well as minimal peptides introduced by Davie et al. [26] appeared together with many others during the last decade; they all fall into this class of catalysts. These structures are highly modular and can be readily modified and finely tuned. Many organocatalysts have been recognized to be reminiscent of natural enzymes in their mode of action and substrate interaction/activation [27]. Their use in several bioinspired methodologies has led to envisaging efficient synthetic routes leading among the others to the obtainment of target compounds of interest in medicinal chemistry.

**Scheme 1:**
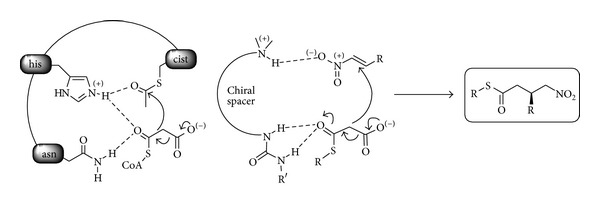
Activation of MAHT in the active of PKA synthase and in the chiral core of the organocatalyst.

In the biosynthesis of fatty acids and polyketides the active site of polyketide synthase (PKS) clearly highlights the key role displayed by hydrogen bonds (Scheme 1) [28].

The possibility of mimicking the hydrogen bond interactions of PKS's with a simple organic molecule (“hunt for smallest enzymes” according to Schreiner) has been shown feasible by using double hydrogen bond-based organocatalysts. The potential of this strategy has been appreciated by synthetic chemists for many years and has been widely used in asymmetric catalysis [29].

**Scheme 2:**
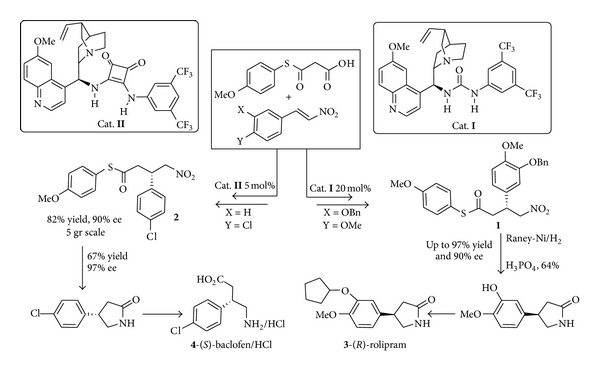
Synthesis of GABA receptors via hydrogen bonds directed organocatalysis mimicry of polyketide synthase.

The application of the enantioselective decarboxylative reaction of malonic half thioesters (MAHTs) to the synthesis of medicinal targets is exemplified by the synthesis of GABA receptor antagonists** 3** and** 4** using** I** and** II** as the organocatalysts (Scheme 2). The *γ*-nitrothioesters** 1** and** 2** easily achievable through these organocatalytic approaches occurring under mild conditions and tolerating both moisture and air are versatile building blocks for further modifications. Among them the formation of *γ*-butyrolactams by reduction of the nitro group followed by intramolecular cyclization leads to intermediates en route to the antidepressant (*R*)-Rolipram [30]** 3** and to gram scale synthesis and transformation to (*S*)-baclofen·HCl** 4,** a GABA receptor antagonist used in the treatment of spasticity [31].

**Scheme 3:**
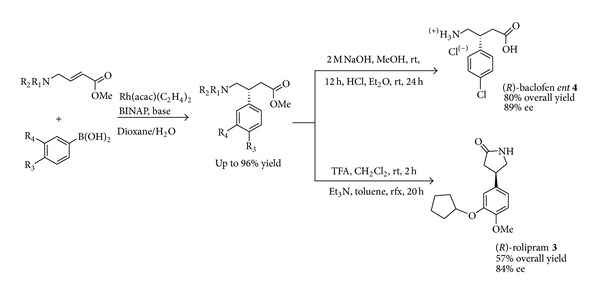
Metal-catalysed synthesis of GABA receptors.

Several enantioselective syntheses of GABA receptors have been reported based on the use of a metal/ligand assembly as the catalyst system. So far (Scheme 3) the Rh(acac)-catalyzed asymmetric 1,4-additions of arylboronic acids to 4-aminobut-2,3-enoic acid derivatives led to (−)-(*R*)-baclofen* ent* **-4** and to (−)-(*R*)-rolipram** 3** in high yields and excellent enantioselectivities [32]. However the use of inert atmosphere (argon) and the to some extent difficult purification by flash chromatography prevent this methodology to be easily applied on large scale.

**Scheme 4:**
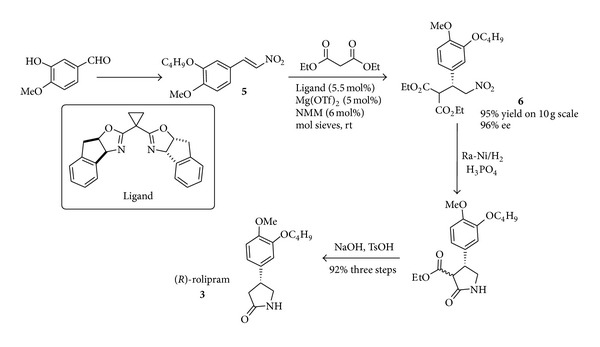
Scalable metal-catalysed synthesis of both enantiomers of rolipram.

An alternative metal-catalysed system [33] in which the potential for scale-up is clear is shown in Scheme 4 and appears highly competitive with the organocatalysed approach. The chiral Lewis acid-catalyzed Michael addition of diethyl malonate to fully elaborated nitrostyrene** 5** allows the nitroester** 6** that upon reduction and saponification leads to the target compound** 3**. Both enantiomers of rolipram** 3** can be accessed in a total of six steps and at 10 gram scale with excellent overall yields of 76% and without chromatography.

The use of magnesium is preferable to many other metal catalysts since toxicity issues are avoided. The dependence on solvents such as chloroform does, however, raise in this method toxicological and environmental issues.

**Scheme 5:**
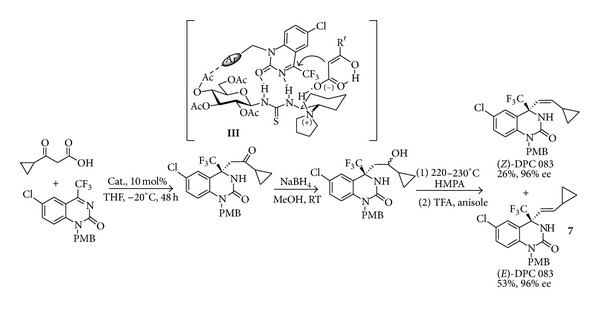
Hydrogen-bonding assembly between organocatalyst, ketimine, and *β*-ketoacid in the preparation of the anti-HIV drug DPC 083.

The range of applications of bioinspired decarboxylative reactions is witnessed by the very recent [34] hydrogen-bond directed enantioselective decarboxylative Mannich reaction of keto acids with ketimines. Under the action of saccharide-derived amino thioureas as chiral catalysts (**III**), this reaction that can be run on a gram scale without any detriment on the reaction outcome leads to the expected trifluoromethylated 3,4-dihydro-quinazolin-2(1*H*)-one rings in very high yields and up to 99% ee. The potential application of this decarboxylative Mannich reaction in the domain of pharmaceutics is demonstrated in a new and efficient and shortcut synthesis of the anti-HIV drug DPC083** 7** shown in Scheme 5. Herein the crucial role of the hydrogen bond interactions in building a complex rigid architecture responsible for the high stereoselectivity is highlighted.

**Scheme 6:**
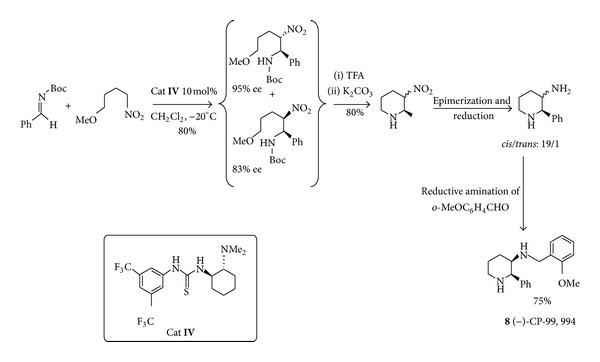
Organocatalytic synthesis of CP-99,994** 8**, a neurokinin-1 receptor agonist.

Hydrogen bond-based organocatalysis also plays a primary role in the synthesis of low molecular weight drug candidates. The aza-Henry reaction (nitro-Mannich reaction) was used by Xu and coworkers [35] for the short asymmetric synthesis of the chiral piperidine derivative CP-99,994** 8** (Scheme 6). The previous asymmetric syntheses of this potent neurokin-1 receptor antagonist were mainly based on the use of metal complexes as catalysts but suffered from several drawbacks, for example, low overall yield and enantioselectivity or a lengthy synthetic route. Notably the organocatalysed Takemoto's synthesis proceeded in five steps without the need to separate the diastereomeric intermediates that were cyclized as a mixture. The catalyst employed was a chiral thiourea** IV**, which served as an activator of both the nitroalkane and imine reactants. The transition state is relatively complex and is dominated by hydrogen-bonding interactions.

**Figure 4:**
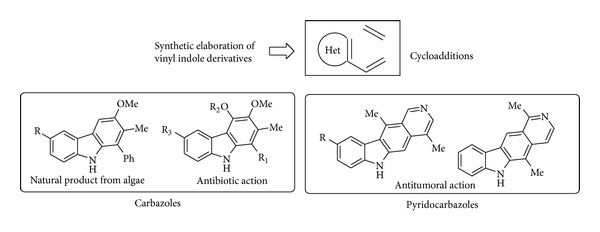
Biological activity of ring-fused indoles.

**Scheme 7:**
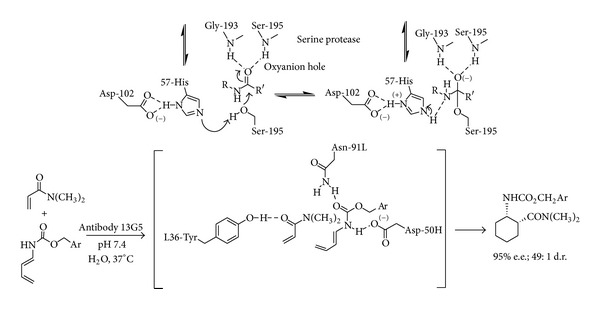
Occurrence of the oxyanion hole in enzymatic processes.

The simultaneous donation of two hydrogen bonds has also proven to be a highly successful strategy for electrophilic activation in enzymes with an “oxyanion hole” having a postulated role in the stabilization of many high-energy tetrahedral intermediates [36]. It appears that living systems discovered and made use of these interactions in the ubiquitous useful ring-forming Diels-Alder reaction eons ago for the construction of complex natural products so that the prospect of discovering a Diels-Alderase mimic would be especially exciting. Following this concept and inspired by the antibody 13G5-catalyzed Diels-Alder cycloaddition of acrylamide with a carbamate (Scheme 7), taking place via a cooperative multiple hydrogen bond coordination to both diene and dienophile [37]; a catalytic asymmetric cycloaddition of 3-vinylindoles with activated dienophiles has been recently reported. The synthetic elaboration of vinyl indole derivatives via cycloaddition appears highly promising in that it leads (Figure 4) to fused poly-heterocyclic ring systems otherwise not easily accessible like carbazoles and pyridocarbazoles with antibiotic and antitumor activities.

**Scheme 8:**
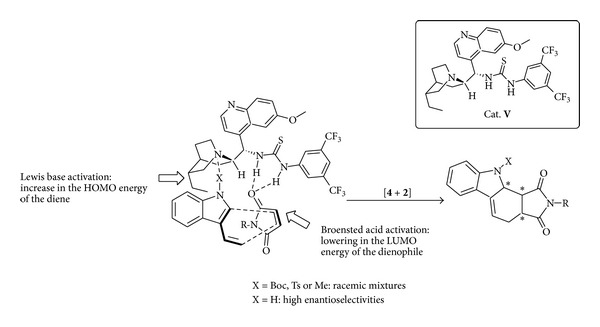
Bifunctional activation in the Diels-Alder reaction of 3-vinylindoles.

**Scheme 9:**
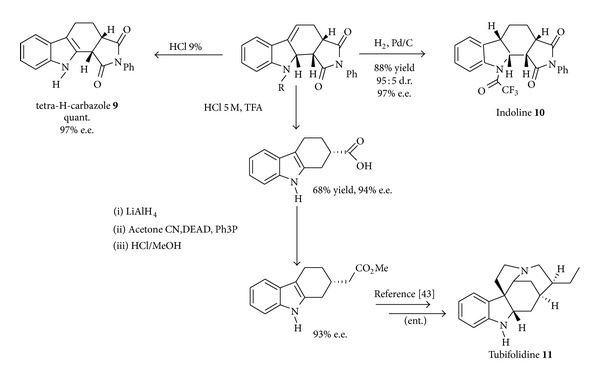
Synthetic elaborations of the vinylindole cycloadducts.

A scenario in which a suitable bifunctional acid-base organic catalyst (**V**) coordinates through H-bond interactions both diene and dienophile leading (Scheme 8) to a highly organized transition state has been designed [38] delivering in very high yields and excellent enantioselectivities a wide range of indolines and tetracarbazoles common scaffolds in a variety of biologically active and pharmacologically important alkaloids [39–41]. The synthetic potential of the cycloadducts is exemplified by the access to indoline** 9**, to tetrahydrocarbazole** 10 **with potent activity against human papillomaviruses [42] and to a precursor [43] of tubifolidine** 11 **a Strychnos alkaloid previously prepared using a nine-step synthesis (Scheme 9).

**Scheme 10:**
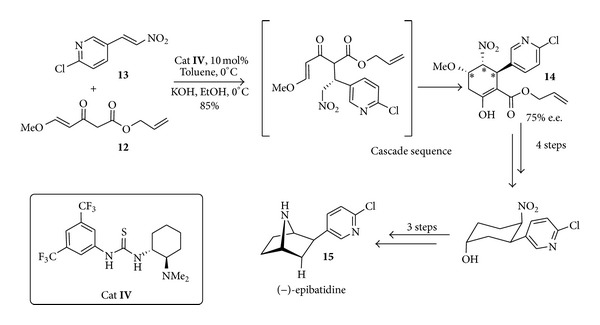
Organocatalyzed cascade synthesis of (−)-epibatidine** 15**.

The combination of hydrogen bond-based organocatalysis and cascade reactions or one-pot processes in the synthesis of therapeutics is powerful and can be illustrated by the synthesis of the alkaloid (−)-epibatidine, developed by the Takemoto's group and based on an enantioselective double Michael addition [44]. The bifunctional thiourea-based organocatalyst** IV** catalysed the first Michael addition of the *γ*,*δ*-unsaturated *β*-ketoester** 12** to the nitroalkene** 13** and on addition of KOH the newly formed nitroalkane cyclized to form the polysubstituted cyclohexene** 14** in a high yield and 75% ee (Scheme 10). The total synthesis of (−)-epibatidine** 15** was achieved in further seven steps from** 14**. Though due to its high toxicity (200 times more potent than morphine) and lacking of selectivity on nicotinic receptors (−)-epibatidine cannot be considered a lead for pharmaceutical development; it has already opened the route to a wide series of more selective and promising derivatives.

Other laboratory scale syntheses based on the use of thiourea-derived bifunctional organocatalysts have been reported leading to targets of interest in medicinal chemistry. Among them a further highly enantioselective (99% e.e.) synthesis of (*R*)-rolipram and of (*3S-4R*)-paroxetine (see Section 2.2.1.) has been accessed through the use of a combined thiourea-cinchona catalyst [45] using a highly enantioselective Michael addition of malonate nucleophiles as key steps. An indanol-thiourea organocatalyst resulted on the other hand very effectively in one of the first enantioselective Friedel-Crafts alkylations of indole with nitroalkenes leading after a synthetic elaboration of the alkylation products to the synthesis of 1,2,3,4-tetrahydro-*β*-carbolines [46] with anti-inflammatory and anti-arrhythmic activities.

#### 2.1.2. Phase Transfer Catalysis

Phase transfer catalysis (PTC) has long been recognized as a versatile catalytic methodology for organic synthesis in both industry and academia. It features operational simplicity, typically mild reaction conditions, inexpensive and environmentally benign reagents and solvents, and relatively cheap catalysts that can be found in reasonable abundance [47]. Moreover, it has proven particularly viable for large- and industrial-scale applications. Chiral phase transfer catalysis has seen an explosive growth in the past couple of decades [48–50] and is still one of the hottest research areas in asymmetric noncovalent organocatalysis [51, 52]. The development through the years of various types of chiral phase transfer catalysts relying on the molecular design of both natural product-derived and purely synthetic quaternary ammonium salts delivered [53, 54] not only higher reactivity and stereoselectivity but also new synthetic opportunities [55]. So far a wide variety of highly enantioselective transformations catalyzed mainly by cinchona alkaloids or binaphthyl-derived quaternary ammonium salts have been introduced and applied to the asymmetric synthesis of biologically active compounds including a number of pharmaceuticals. Furthermore pharmaceutical companies have demonstrated the viability of asymmetric phase transfer reactions in the large-scale preparation of drugs.

**Scheme 11:**
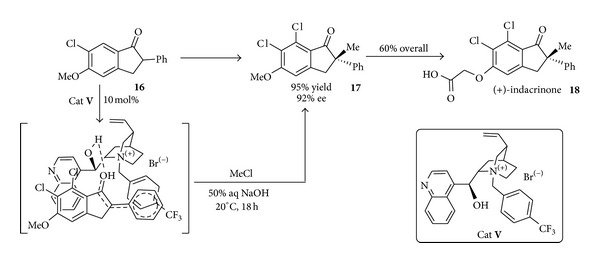
Phase transfer catalysed synthesis of (+)-indacrinone** 18**.

Interestingly the first landmark example in the domain of chiral phase transfer organocatalysis was developed by Merck as early as in 1984 for the synthesis of a uricosuric drug (+)-indacrinone (MK-0197). In this work [56] the highly enantioselective alkylation of compound** 16** was achieved using the cinchona alkaloid derivative** V** (obtained by N-alkylation of the quinuclidine core), NaOH as a base, and MeCl as the alkylating agent (Scheme 11). Using this approach intermediate** 17**, used for the synthesis of the indacrinone** 18**, could be accessed in high yield and enantiomeric purity on a pilot plant scale (~75 Kg); the cost of producing this enantiomer is significantly lower than the cost of producing the same molecule by a resolution process.

Studies on the origin of the stereoselectivity substantiated the hypothesis of a tight ion pair transition state where the enolate anion and the cationic catalyst were held close to each other through *π*-interactions.

**Scheme 12:**
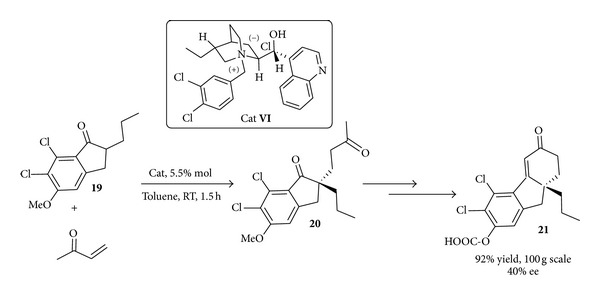
Synthesis of a drug candidate for treatment of brain edema via PTC catalysis.

Almost in the same period scientists from Merck demonstrated that cinchona derivatives such as** VI** could catalyse the Michael addition of ketone** 19** with methyl vinyl ketone (MVK) under mild conditions and crucially, at large scale [57] (Scheme 12) to give** 20**.

The ultimate goal of this study was the synthesis of drug candidate** 21** (and analogues) for the treatment of brain edema and traumatic head injuries [58]. This reaction was carried out under various conditions and the operationally simple liquid/solid system gave excellent isolated yields at 100 g scale albeit with modest levels of enantioselectivity. These early examples showed the potential power of the asymmetric PTC reactions for industrial-oriented synthesis.

**Scheme 13:**
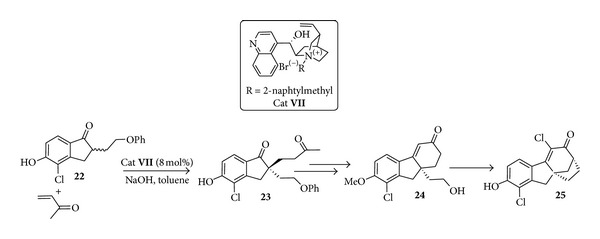
Pilot-scale synthesis of an estrogen receptor-*β*.

The learning generated in the previous examples was of great benefits for further developments of chiral phase transfer organocatalysis. An impressive use of the use of quaternary salts of cinchona alkaloids in phase transfer catalysis for the pilot scale production of drug candidates is shown in the development at Merck Sharp & Dohme of the asymmetric synthesis of an estrogen receptor *β*-selective agonist [59] (Scheme 13). The base-catalysed Michael addition of the enolate of indanone** 22** to MVK, in the presence of a (+)-cinchonine-derived quaternary ammonium phase transfer catalyst** VII**, gives diketone** 23** in enantioenriched form. Robinson annulation then follows with construction of the cyclohexenone ring of tetrahydrofluorenone** 24** that upon cyclization gives rise to the expected target** 25**. Overall the chemistry developed has been used to prepare >6 kg of the drug candidate in 18% overall yields and with >99% ee. The 2-naphtylmethylcinchoninium bromide catalyst** VII** selected on the basis of the 50% ee in the Michael addition step and on the bulk commercial availability of the required 2-naphtylmethyl bromide and the agitation rate were parameters critical to the success of this reaction.

**Scheme 14:**
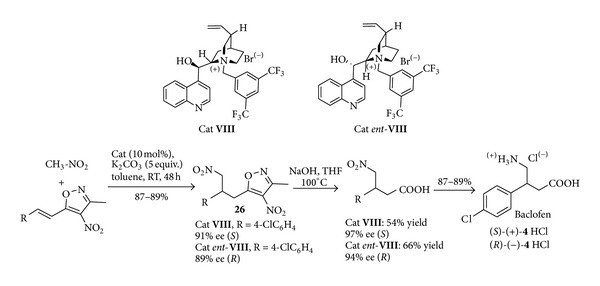
Laboratory-scale synthesis of both the enantiomers of baclofen** 4**.

In another more recent example the capability of chiral phase transfer catalysis based on quaternary ammonium salts** VIII** and* ent*-**VIII**-derived from cinchona alkaloids to induce highly enantioselective C–C bond forming reactions has been disclosed in the conjugate addition of nitroalkanes to 4-nitro-5-stirylisoxazoles, a valuable synthetic alternative to cinnamic esters [60] (Scheme 14). The transformation of the Michael adducts** 26** into *γ*-nitro acids could be easily performed, and the subsequent Raney-Ni reduction gave the hydrochlorides of the GABA receptors (*S*)- and (*R*)-baclofen** 4** thus outlining a short organocatalysed route alternative with respect to that outlined in Scheme 1.

The accessibility of both the enantiomers in good yields and excellent enantioselectivities, the wide reaction scope, and the easy availability and the use of inexpensive organocatalysts outline major assets of this organocatalysed methodology.

#### 2.1.3. Lewis and Brønsted Base Catalysis

**Figure 5:**
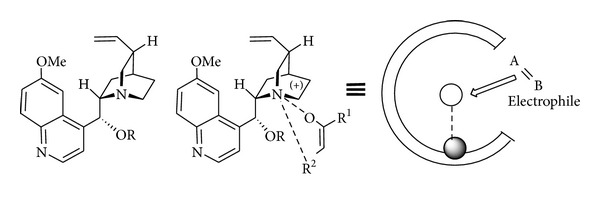
Cinchona alkaloids catalysis through chiral contact ion pair.

Nucleophilic catalysts have had a wide role in the development of new synthetic methods [61]. In particular, the cinchona alkaloids catalyse many useful processes with high enantioselectivities [62]. They can be used as bases to deprotonate substrates with relatively acidic protons such as malonates, forming a contact pair between the resulting anion and the protonated amine. This interaction leads to a chiral environment around the anion and permits enantioselective reactions with electrophiles (Figure 5).

Since the seminal publication by Hiemstra and Wynberg [63] there have been different applications of this methodology with significantly improved catalysts [64]. Important in many of these processes is the ability to control the formation of quaternary centers with high enantiomeric excess [65]. The robustness and the easy availability of the commercially available cinchona derivatives attracted in the last decades increasing interest of both the academic and applied research. In medicinal chemistry relevant targets such as anticancer and antiparasitic agents were approached by using this methodology.

**Scheme 15:**
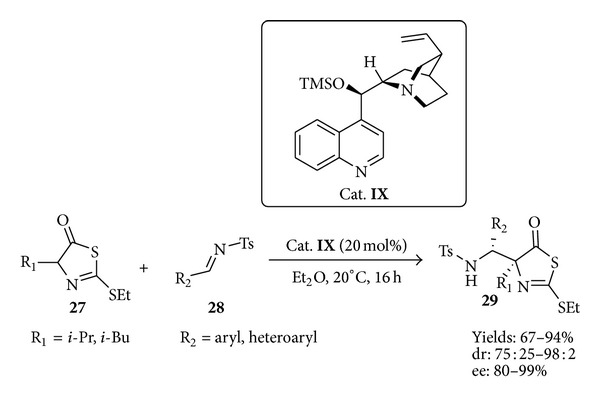
Synthesis of anticancer thiazolone derivatives by organocatalytic aza-Mannich reaction.

In the past 10 years, the number of chiral, nonracemic pharmaceuticals on the market was consistently increasing and many new single enantiomer drugs were produced to offer enhanced therapy and reduced toxicity. Organocatalysis emerged to be an effective way to reach this goal. A series of chiral 2-ethylthio-thiazolone derivatives** 29** have been prepared (Scheme 15) by a straightforward enantioselective aza-Mannich addition of thiazolones** 27** to N-tosylimines** 28** catalyzed by a simple cinchona alkaloid (**IX**) as the chiral base with a 20 mol % of catalyst loading using diethyl ether as solvent [66]. The derivatives bearing a quaternary center were obtained in good yields and, in general, with high diastereo- and enantioselectivities. All the compounds, evaluated in five human cell cancer lines using MTT essay, caused a dose-dependent growth inhibitory effect on all the tested cancer lines. This study provides a foundation for further developments of new single enantiomer anticancer drugs.

**Scheme 16:**
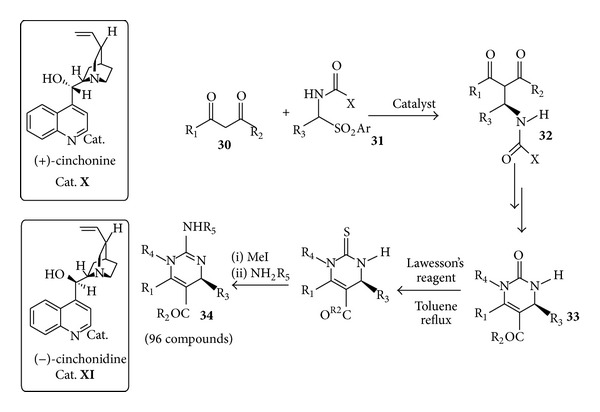
Synthesis of a library of dihydropyrimidinones** 34** anti-malarial derivatives by a cinchona alkaloid-driven key organocatalytic step.

Malaria is one of the most important diseases of the third world and the efficacy of the available drugs is limited by emerging resistance. In 2011, in an extensive effort to find unique chemotypes for the treatment of malaria, it has been found that dihydropyrimidinone-derived guanidine derivatives were the most promising [67]. These guanidine analogs** 34** were synthesized in a multistep synthesis with commercially available and inexpensive (+)-cinchonine** X** and (−)-cinchonidine** XI** promoting the key organocatalytic step (Scheme 16).

In this step, the diketone derivative** 30** was deprotonated by the nitrogen of the chiral base (cinchonine or cinchonidine) which attacks the imine formed* in situ* starting from** 31** to give the corresponding intermediates** 32** in high enantiomeric excesses. These were then cyclised into dihydropyrimidinones** 33**. Being the two organocatalysts pseudoenantiomers, both enantiomers of dihydropyrimidinones could be synthesized. Further treatment of** 33** with Lawesson reagent, followed by sulphur alkylation and its substitution with different anilines led to a library of 96 guanidine derivatives** 34**.

**Scheme 17:**
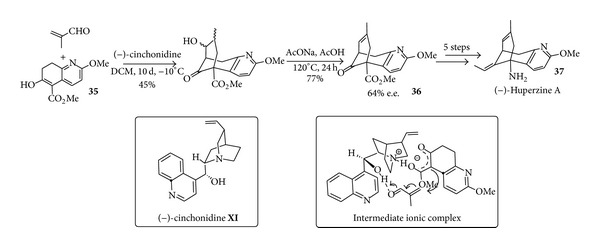
Preparation of (−)-huperzine A by means ofan organocatalysed Michael/aldol cascade reaction sequence.

Another quite impressive example of how simple and unmodified cinchona alkaloids can be used for the synthesis of medicinally important scaffolds is provided by the synthesis of (−)-uperzine A** 37** currently being tested in clinical trials as a promising drug for the treatment of Alzheimer disease [68]. This reaction that can be considered as the first application of cascade reaction to the synthesis of targets in medicinal and natural product chemistry dates back to 1998 when the field of organocatalysis was just at its infancy. Huperzine-A, containing a challenging bridged tricyclic core, was obtained via a simple Michael/aldol cascade reaction sequence between a *β*-ketoester** 35** and methacrolein (Scheme 17). The commercially available and inexpensive organocatalyst (−)-cinchonidine (**XI**) acts as a bifunctional organocatalyst. As a base it deprotonates** 35** forming a chiral ion pair but the secondary alcohol function of the catalyst simultaneously activates a methacrolein molecule by forming a distinct hydrogen bond and incorporating it into the ionic complex. The Michael reaction, as the first step of the cascade reaction is thus initiated, followed by intramolecular aldol condensation. The tricyclic core** 36** of (−)-huperzine A was formed with an overall yield of 60% and 64% enantiomeric excess (e.e.). The completion of the total synthesis starting from** 36** required 5 further steps. It is worth noting that the synthesis of* ent*-**37** could be achieved in the same way starting from cinchonine. Though to some extent disappointing for the modest enantioselectivity this procedure outlines a rapid one-pot entry to molecular complexity by using a simple metal-free, commercially available, and inexpensive air- and moisture-stable organocatalyst.

#### 2.1.4. Brønsted Acid Catalysis

Recently chiral Brønsted acids have found widespread application in organocatalysis [69, 70]. For instance, in one of the most relevant processes the action of a Hantzsch ester, a biomimetic source of hydride, combines with that of chiral phosphoric acid as the catalyst. This can be considered as a metal-free simple H(+)-H(+) cascade reaction and has become a favourite application to the enantioselective reduction of nitrogen-containing heterocycles like pyridines or quinolines to the corresponding tetrahydroquinolines and tetrahydropyridines [71, 72]. This approach gives access to a variety of highly enantioenriched heterocycles that are privileged structures in natural products and drugs.

**Scheme 18:**
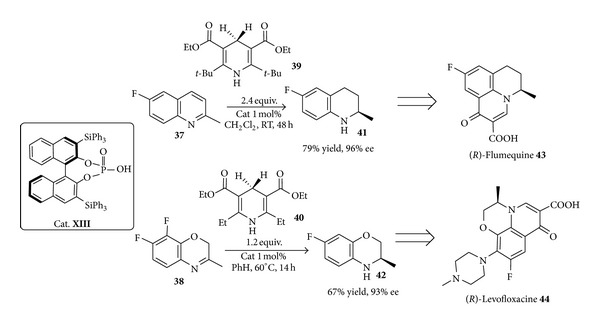
Enantioselective transfer hydrogenation for the preparation of tricyclic fluoroquinolone antibacterial agents** 43** and** 44**.

The preparation of fluoroquinolones reported by Rueping and coworkers [73] outlines the application of the transfer hydrogenation process to the synthesis of building blocks that have been utilized to complete the metal-free synthesis of drugs like (*R*)-flumequine (**43**) or (*R*)-levofloxacin (**44**) that display antibacterial activity towards a broad spectrum of bacteria [74, 75]. The readily available fluorinated quinoline** 37** and benzoxazine** 38** were reduced in the presence of Hantzsch esters** 39** or** 40** with only 1 mol% of the sterically demanding chiral phosphoric acid** XII** as catalyst to give the corresponding hydrogenated compounds** 41** and** 42** in very good yields and with excellent enantioselectivities (Scheme 18).

The synthesis of the two targets** 43** and** 44** was then accomplished in three more steps.

**Scheme 19:**
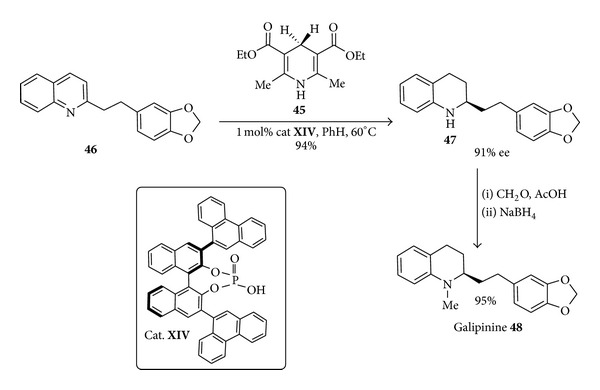
Synthesis of (+)-galipinine via binolphosphoric acid-catalyzed enantioselective cascade reduction.

Moreover through the use of only 1 mol% of the binaphthol phosphate catalysts** XIV**, a stepwise hydride transfer from the Hantzsch ester** 45** to quinoline** 46** afforded [76] the corresponding tetrahydroquinoline** 47** in excellent yields and enantioselectivities (Scheme 19). Mechanistically it has been assumed that this enantioselective cascade hydrogenation occurs in two cycles involving iminium ion an enamine species, respectively. A reductive N-methylation concludes a concise synthesis of (+)-galipinine** 48** showing antimalarial activity on Plasmodium Falciparum for the chloroquine-resistant strains.

**Scheme 20:**
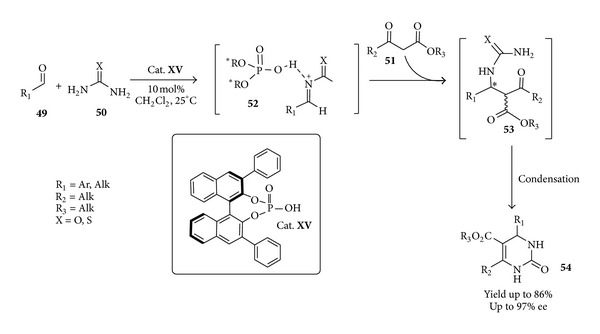
Enantioselective chiral Brønsted acid-catalyzed three-component Biginelli reaction.

Another remarkable and to some extent different use of a chiral phosphoric acid in the synthesis of a drug candidate is represented by the one-pot acid-catalyzed three-component condensation of an aldehyde** 49**, a thiourea** 50,** and a *β*-ketoester** 51** in an asymmetric Biginelli reaction to give the chiral 3,4-dihydropyrimidin-2-one derivatives** 54 **[77]. These scaffolds are privileged structures that depending on the substitution pattern exhibits a variety of important pharmacological properties like the inhibition of Hepatitis B virus replication. Here the chiral phosphoric acid** XV** catalyzes the Biginelli reaction by forming a chiral N-acyliminium phosphate ion pair** 52** to which enantioselective addition of *β*-ketoesters** 51** occurs to generate optically active** 54** *via* the enantioenriched intermediate** 53** (Scheme 20).

An asymmetric variant with an ytterbium-based catalyst for this Biginelli reaction was reported earlier [78], but the discovery of a metal-free synthesis by using Brønsted acid** XV**, which avoided contamination of the product with traces of metal, resulted in an important advancement. The phosphoric acid-based catalyst matched or even improved the level of conversion and stereoselectivity of the corresponding Lewis acid-catalyzed reaction, while maintaining the same substrate scope.

### 2.2. Covalent Organocatalysis

The area of amine-organocatalysed reactions is clearly dominated by secondary amines due to the versatility of possible combination of enamine (EN) and iminium (IM) activation. However, the primary amino function as a part of a chiral scaffold could be engaged as well in a number of synthetically appealing organocatalysed reactions. Several reviews on amino catalysis have recently appeared [79, 80].

#### 2.2.1. Secondary Amine Organocatalysis via Enamines and Iminium Ions

The reaction that alerted the scientific community to the potential of organocatalysis was a proline-catalysed intramolecular aldol reaction reported almost simultaneously by two groups during the early 1970s [81, 82]. It was not until List et al. published a related intermolecular process [83] that secondary amine catalysis via enamine, inspired by Nature's aldolase enzymes, became* en vogue* in the domain of organocatalysed reactions. Since this report, there have been many subsequent publications of catalytic reactions via enamines. Proline-catalysed Mannich reactions [84], dihydroxylations [85], cross aldolizations [86], and aminations [87, 88] have held persistent interest in the area of asymmetric catalysis.

**Scheme 21:**
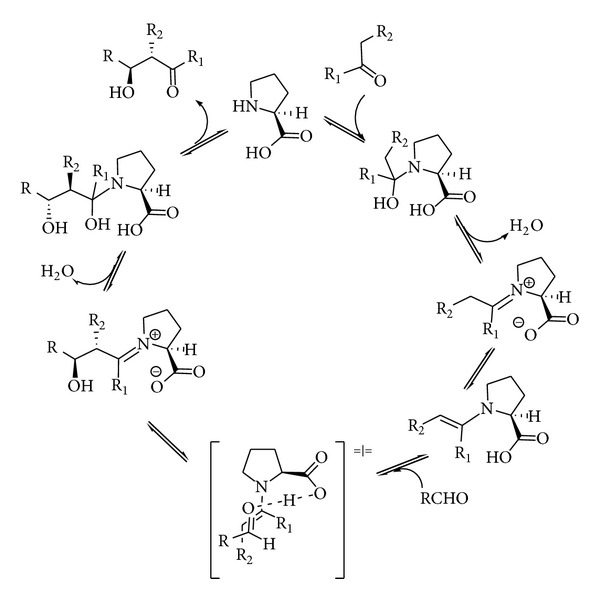
Mechanism for the proline-catalysed intermolecular aldol reaction.

Mechanistically, this enamine catalysis might be better described as a bifunctional catalysis because the amine-containing catalyst (proline) typically interacts with a ketone substrate to form an enamine intermediate but simultaneously engages with an electrophilic reaction partner through either hydrogen bonding or electrostatic interaction (Scheme 21).

**Scheme 22:**
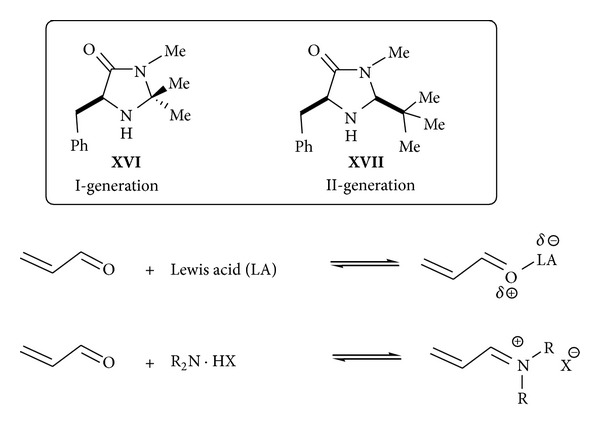
Iminium activation through LUMO lowering.

The capacity of chiral amines to function as enantioselective LUMO-lowering catalysts for a range of transformations that had traditionally employed Lewis acids has also been extensively used in organocatalysis. This strategy, termed iminium activation, was founded on the mechanistic postulate that the reversible formation of iminium ions from *α*,*β*-unsaturated aldehydes and chiral amines might emulate the equilibrium dynamics and *π*-orbital electronics that are inherent to Lewis acid catalysts, thereby providing a platform for designing organocatalytic processes (Scheme 22). The first generation catalyst to fulfil criteria such as efficient and easily reversible iminium ion formation, discrimination of the olefin *π*-face, and easy preparation was imidazolidinone** XVI** that in 2001 evolved in the more efficient imidazolidinone catalyst** XVII** (second generation). With its tailor-made family of imidazolidinone catalysts, iminium catalysis has been successfully applied to a broad range of chemical transformations including cycloadditions [89, 90], conjugate additions [91–93], hydrogenations [94], and cascade reactions [95]. The operational simplicity of these processes made them attractive alternatives to Lewis acid catalysis.

A number of drugs currently on the market have been approached with the enamine-iminium-based organocatalysis taking advantage by the simplicity of these inexpensive organocatalyst and by their high efficiency.

The case of warfarin is a very good example of the exceeding utility of organocatalytic methodologies in the assembly of relatively simple yet highly relevant molecules and many iminium-based organocatalysed processes have been designed for this aim. Warfarin is a vitamin K analogue, inhibiting vitamin K epoxide reductase. Its sodium salt, commercialised mainly under the trade names Coumadin and Marevan, is one of the most widely prescribed anticoagulants. Warfarin has been administered as a racemate for over fifty years; however, its two enantiomers display remarkably different pharmacological and pharmacokinetic profiles. Even if the* S* isomer shows higher activity, it is metabolised more rapidly than its less active* R* counterpart [96]. Thus, production of both (*R*)- and (*S*)-warfarin in enantiopure form might be of importance for a tailored patient treatment [97].

An obvious synthetic approach to warfarin is represented by the Michael addition of 4-hydroxycoumarin to benzylideneacetone, a reaction which is well posited for iminium ion catalysis through enone activation. Such an approach appears superior and more straightforward compared to the few reported catalytic asymmetric methods based on organometallic chemistry, which rely on more tortuous oxidation-reduction sequences with protecting groups usage [98, 99]. Accordingly, the feasibility of the organocatalytic strategy leading directly to warfarin has been well demonstrated, with the large number of reports well witnessing its success and appeal. A survey of the various organocatalysts engaged in this synthesis and of the related results is shown in Table 1.

The first approach to enantioenriched warfarin through iminium ion catalysis, disclosed in 2003, involved [100] the employment of the phenylenediamine-derived catalyst** XVIII** and provided after prolonged reaction time the target compound in good yield and moderate enantioselectivity, which could be improved to >99% by a simple crystallisation (Table 1, entry 1). It was also mentioned [101] that the reaction could be performed on a kg scale by employing a related catalyst structure, recoverable after reaction completion. The approach is the same as the commercial synthesis of Coumadin in a retrosynthetic sense, but crucially the presence of imidazolidine catalysts** XVIII** affords an enantioselective reaction whilst the commercial synthesis is racemic. A few years later it was discovered that the putative catalyst** XVIII** was undergoing a hydrolysis under the reaction conditions, delivering the corresponding free phenylenediamine, which was the actual catalytic species of the Michael addition. On this basis, the results obtained in this transformation could be improved [102] by employing a related diamine** XIX** in combination with a large amount of an acidic cocatalyst (acetic acid, entry 2). Subsequent efforts with simple primary diamine catalysts were directed [103] at improving the practical applicability of this reaction and included the discovery that the catalyst loading of phenylenediamine** XX** could be lowered by exploiting a combination of Lewis (lithium perchlorate) and Brønsted (acetic acid) acid cocatalysts in the reaction (entry 3) and that water as reaction medium under ultrasound activation [104] provided a fast protocol allowing the isolation of warfarin in essentially enantiopure form by simple filtration of the reaction mixture (entry 4). Variations in the phenylenediamine motif by functionalization of one of the amines have also been reported and include the monoamine** XXI**, generated* in situ* by acidic hydrolysis from a corresponding diimine [105], and the monophosphonamide** XXII** [106].

**Figure 6:**
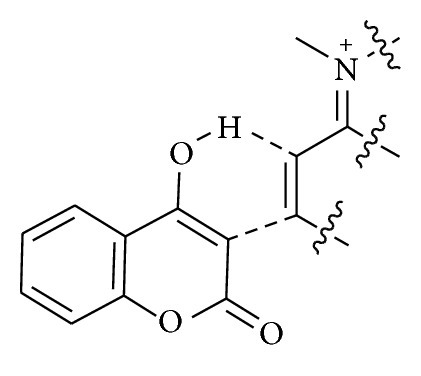
Reaction between coumarin and iminium ion in a concerted pathway.

Both of these catalysts, employed in combination with carboxylic acids, provide the product warfarin in excellent enantioselectivity (entries 5 and 6). A different diamine, namely, 1,2-cyclohexanediamine, was employed for the preparation of catalyst** XXIII** bearing a lithium sulfate functionality on one of the amines, which however led [107] to rather poor results (entry 7). The lithium based catalyst** XXIII **was designed to exploit the mild Lewis acidity of lithium for the intramolecular activation of the imine formed between the catalyst and the Michael acceptor. Interestingly, computational studies showed that a related reaction (with cyclohexenone as Michael acceptor) follows an ene-type concerted pathway, with simultaneous C–C bond formation and proton transfer between the hydroxyl coumarin and the iminium ion activated olefin of the Michael acceptor (Figure 6).

A structurally distinct primary amine catalyst** XXIV**, derived from a Cinchona alkaloid, was also successfully applied in this reaction [108] with very good results at relatively high catalyst loadings (entry 8). Highlighting the high interest that this specific transformation has surged in the academic community, a recent publication reported the search for a more readily available catalytic system, considering that the preparation of the phenylenediamine cores of catalysts** XVIII**–**XXII** is rather troublesome and that the synthesis of the Cinchona derivative** XXIV** presents considerable safety issues (it is prepared through a Mitsunobu reaction which employs hazardous azides). To that purpose, the focus was set [109] on amino acid as starting materials, resulting in the disclosure of the diphenyl phenylglycinol** XXV** as a moderately efficient and easily available catalyst for the obtainment of warfarin in enantioenriched form (entry 9). The same report highlighted the better performances of primary amine compared to secondary amines in this reaction, reporting also the poor performances in this transformation of other popular secondary amine catalysts, such as proline or a MacMillan imidazolidinone. However, it was more recently demonstrated [110] that also a secondary amine, the dimeric proline-derived catalyst** XXV**, could be applied in this Michael addition reaction with fairly good results (entry 10).

**Scheme 23:**
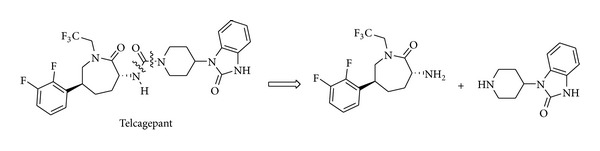
Retrosynthetic disconnection of Telcagepant.

Simple iminium ion catalysed reactions have also been inserted into more complex multistep reaction sequences; some of them herein are discussed in detail leading to medicinally relevant compounds. In this context, the example of Telcagepant is significant, as an iminium ion catalysed reaction was selected as the key step for the establishment of the stereochemistry in an industrial-scale preparation of this molecule. Telcagepant [111] is an antagonist of the calcitonin gene-related neuropeptide and was considered highly promising for the treatment of migraine avoiding concomitant cardiovascular problems often generated by other antimigraine agents. Telcagepant can be retrosynthetically disconnected between a chiral 2-amino-5-aryl caprolactam and a 4-aminopiperidine units, assembled with concomitant urea formation (Scheme 23).

**Scheme 24:**
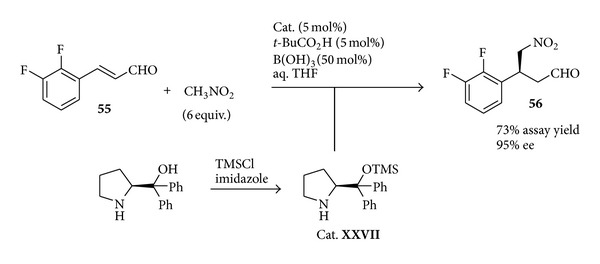
Organocatalysed Michael addition.

A first-generation multi-kg (>500) process [112], relying on a dynamic kinetic resolution of the enantiomers through crystallisation, showcased several pitfalls in the preparation of the chiral caprolactam, thus calling for alternative synthetic strategies to this unit. To this end, an organocatalytic approach, namely, an iminium ion catalysed conjugate addition of nitromethane to a proper cinnamaldehyde [113], was judged as more promising than other asymmetric preparations based on transition metal catalysed reactions, such as ruthenium-catalysed hydrogenation [114] and rhodium based Hayashi-Miyaura addition [115]. Although previously reported with related substrates [116, 117] the organocatalytic step required a careful optimisation for its large scale implementation, as the formation of several by-products was observed under the reported conditions. In particular, to avoid the formation of acetals the usual alcoholic solvents were replaced with a THF/water solvent mixture. The employment of a carefully tailored additive mixture composed by boric and pivaloyl acid proved also to be necessary to achieve an acceptable reaction rate while minimising by-products formation. A final amelioration involved the employment of the crude mixture of the* in situ* silylated diphenylprolinol** XXVII** as catalyst [118, 119], thus avoiding its purification. Finally, the reaction could be performed on a pilot plant (>100 kg) affording the desired Michael adduct** 56** in 73% assay yield and 95% enantiomeric excess (Scheme 24).

**Scheme 25:**
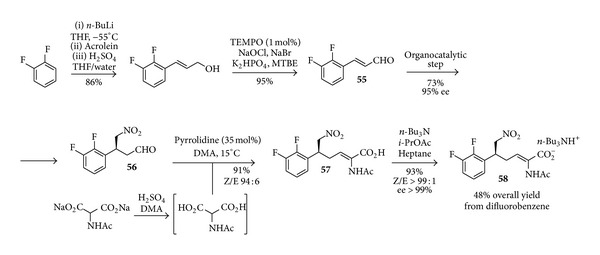
Large scale preparation of** 58**.

Obviously, both the preparation of the starting aldehyde and the subsequent reaction steps leading to the caprolactam had also to be carefully optimised for the large scale synthesis. The first part of the overall sequence is depicted in Scheme 25 and involves the preparation of an allylic alcohol from difluorobenzene through lithiation, quenching with acrolein, acid promoted rearrangement, and TEMPO catalysed oxidation to give the substrate** 55** to be submitted to the organocatalytic step. The aldehyde product** 56** was not isolated, but treated directly in a Doebner-Knoevenagel type reaction with an* in situ* generated acetamido malonic acid (not isolated due to safety issues) in the presence of pyrrolidine. This decarboxylative Knoevenagel-type reaction, even if required extensive optimisation, was preferred over more established Wittig protocols based on atom economy reasoning. The resulting acid** 57** was isolated and upgraded to >99% ee as a tributyl ammonium salt** 58**, obtained in an impressive 48% overall yield starting from difluorobenzene.

**Scheme 26:**
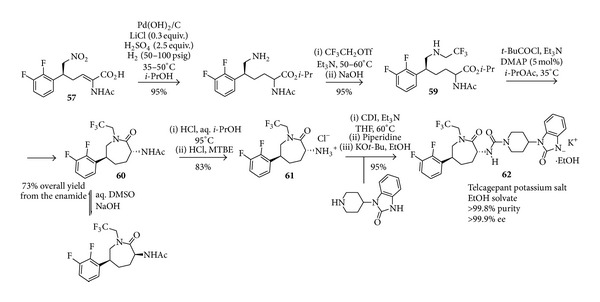
Second part of the synthesis of Telcagepant.

Hydrogenation of this enamide intermediate (**57**) with concomitant nitro group reduction proved to be very challenging, due to the unwanted formation of desfluoro products, which could not be present in more than tiny (0.2%) amounts for the successful employment of this synthesis for telcagepant production. After several attempts (Scheme 26), the hydrogenation reduction showed the desired features when performed on the acid in the presence of lithium salts, in* iso*propanol under acidic conditions, conditions which also promoted esterification of the carboxylate. Trifluoroethyl alkylation with a triflate gave the N-alkylated compound** 59**, whose ester was hydrolysed to the carboxylic acid.

Cyclisation involved the employment of a mixed anhydride, possible for the decreased nucleophilicity of the trifluoroethyl nitrogen, and was followed by a base promoted dynamic crystallisation process in aqueous NaOH/DMSO mixture, which allowed the isolation of the 2-acetamido caprolactam** 60** in 73% overall yield from the enamide. Acidic hydrolysis of the acetamide gave the amine** 61**, which was engaged in urea formation by treatment with carbonyl diimidazole and the corresponding piperidine. Deprotonation of the benzamide hydrogen in ethanol furnished the potassium salt of telcagepant** 62** as an ethanol solvate, with >99.8% purity and >99.9% ee. This environmentally responsible synthesis contains all of the elements required for a manufacturing process and prepares telcagepant** 62** with the high quality required for pharmaceutical use.

The key role played by proline-derived organocatalysts in medicinal chemistry is also witnessed by the recent synthesis of maraviroc. Human immunodeficiency virus (HIV) is a pandemic that was first recognized in 1981. In 2008 HIV caused the death of 2 million people due to the acquired immune deficiency syndrome (AIDS). Due to high medical need the exciting prospect of a new class of drugs for HIV led to the rapid development of the academic and industrial research in this field. Maraviroc (UK-427,857) (**69**) is a chemokine receptor 5 (CCR-5) receptor antagonist that is currently developed at Pfizer for the treatment of HIV. A robust, plant-suitable synthesis of** 69** was urgently required to manufacture larger quantities and the process research [120, 121] of a commercialisable route has been recently developed.

**Scheme 27:**
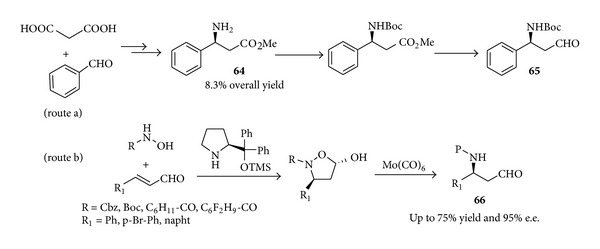
Resolution-based (route a) and organocatalysed (route b) asymmetric synthesis of *β*-amino aldehydes.

The chiral *β*-amino aldehyde** 65** a key intermediate in synthesis of** 68** was produced in the industrial process in an 8.3% overall yield in a multistep sequence in which the enantioenriched *β*-amino ester** 64** was obtained by tartaric acid resolution of the racemic counterpart. Moreover the resolution of the *β*-amino ester was only moderately efficient with two recrystallisations required to achieve the desired enantiomeric excess of >95% (Scheme 27, route a).

An interesting perspective for introducing improvements in this process is offered by organocatalysis. In a recent paper [122] the Córdova group has proposed the asymmetric synthesis of maraviroc and its analogues via an organocatalysed highly enantioselective synthesis of the key chiral *β*-amino aldehyde** 66** fragment. The assembly of** 66** occurs via a two-step protocol involving catalytic enantioselective tandem aza-Michael hemiacetal formation between hydroxylamine and enal followed by N–O cleavage (Scheme 27, route b).

The use of (*S*)-diphenylprolinol silyl ether** XXVII** as the catalyst appears to be very helpful in providing the *β*-amino aldehydes** 66** in yields up to 75% and remarkably good (92–95%) enantioselectivities. Moreover the tolerance of the organocatalysed reaction to a wide range of protecting groups at nitrogen opens the route to structural diversity leading to different analogues of maraviroc of potential interest for scouting new medicinal targets.

**Figure 7:**
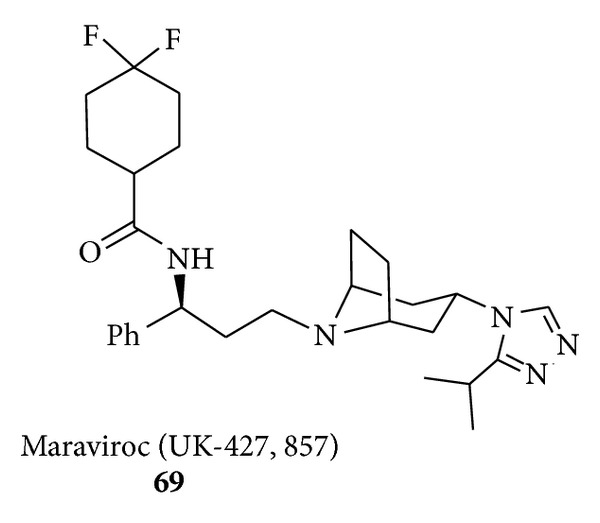
The structure of maraviroc (**69**).

**Scheme 28:**
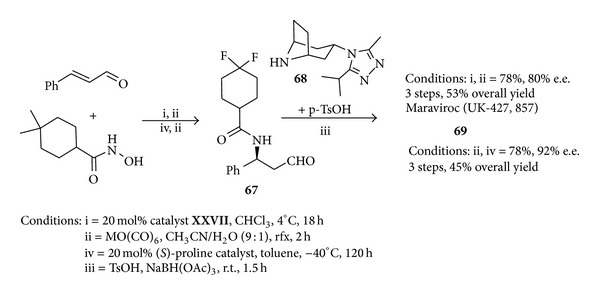
Silylated prolinol-catalysed enantioselective synthesis of Maraviroc.

Following the organocatalysed methodology maraviroc** 69** (Figure 7) could be obtained (3 steps) by reaction of *β*-amino aldehyde** 67** and tropane** 68** in 53% overall yield with 80% e.e. and (3 steps) 45% overall yield with 92% e.e. by varying the reaction conditions (Scheme 28). It is also worth noting that the reaction presented herein can be scaled up. Thus the multigram-scale reaction between cinnamic aldehyde (31.4 g) and Cbz-protected hydroxylamine in the presence of 7 mol% of (*S*)-proline-derived catalyst gave the corresponding 5-hydroxyoxazolidinone precursor of the of *β*-amino aldehyde** 67** in 91% yield (65 g) and 99% e.e.

**Scheme 29:**
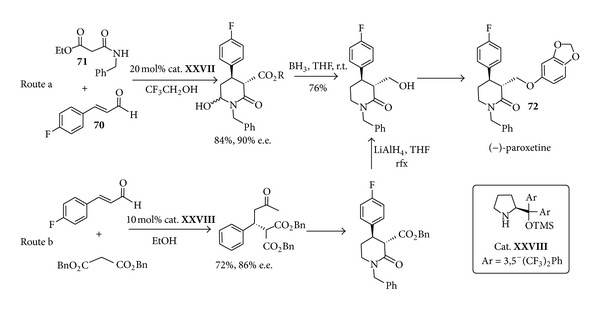
Formal syntheses of (−)-paroxetine via organocatalysis.

Iminium ion organocatalysis appears to be key step in the synthesis of the antidepressants (−)-paroxetine (**72**). This molecule originally marketed by GlaxoSmithKline as Seroxat, a blockbuster selective serotonin reuptake inhibitor for the treatment of depression and anxiety related disorders, has previously been focused on enzymatic asymmetric desymmetrization [123, 124] chiral auxiliary-assisted [125], or asymmetric, deprotonation reactions [126]. In the previous methodologies the total synthesis of this chiral compound consisted of approximately 12–14 steps. A novel approach, based on organocatalysis employing a chiral proline-derived catalyst led to a number of brief (formal) syntheses of paroxetine. Using simple building blocks like fluorocinnamaldehyde** 70** and malonate derivative** 71** with the silylated prolinol** XXVII** as the organocatalyst, Michael reaction was shown (Scheme 29, route a) to proceed in good yields and stereoselectivity [127] to afford the target product** 72** in a three-step sequence. A previous report [128] leading to (−)-paroxetine in six steps overall and based on organocatalyst** XVIII** had also shown the possibility of performing a potentially scalable Michael addition (Scheme 29, route b).

In both cases, the nature of the solvent was highlighted as a key parameter, and polar-protic solvents were required. Whereas the latter example utilised a much more industrially friendly solvent (ethanol versus trifluoroethanol), the reaction times were greater, running for multiple days. Regardless, the potential step saving in these routes is significant taking into account that current industrial syntheses are typically 10–15 steps. Moreover the simplicity of the chemistry involved makes these organocatalysed processes suitable candidates or further scale-up activities.

The spirocyclic motif is featured in a number of natural products as well as medicinally relevant compounds [129–131]. The stereocontrolled construction of the spirocyclic indole core, of high interest for the synthesis of biologically active compounds, poses a challenging synthetic problem mainly in connection with the installation of the spiroquaternary stereocenter. Only a few asymmetric transformations based on cycloaddition processes [132–134] or on the intramolecular Heck reaction [135, 136] have proven successful for achieving this goal. The asymmetric cascade organocatalysis exploiting the ability of chiral amines to combine two modes of activation of carbonyl compounds* via* iminium and enamine catalysis into one mechanistic scheme allows the direct, one-step synthesis of complex spirocyclic oxindoles having multiple stereocenters.

**Scheme 30:**
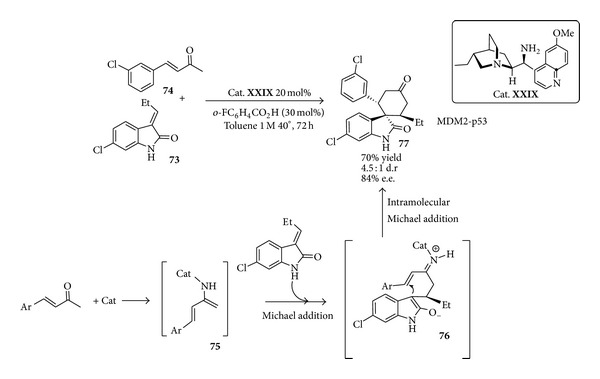
Tandem double Michael addition via enamine-iminium activation sequence toward spirocyclic oxindole.

Among them the synthesis of spirooxindole** 77** recently patented by Hoffmann-La Roche [137], a specific and potent inhibitor of MDM2-p53 interaction and innovative target for the discovery of anticancer agents [138], has been recently reported [139] which highlights the potential of organocascades in building complex structures of interest in medicinal chemistry (Scheme 30). In the one-pot double organocascade compound** 73** would first act as a Michael acceptor, intercepting the nucleophilic diamine intermediate** 75** generated by condensation of the catalyst** XXIX** with the *α*,*β*-unsaturated ketone** 74**. The resulting Michael addition product** 76** would then selectively engage itself in an intramolecular iminium catalysed conjugate addition to afford the spirooxindole** 77**. The target product could be obtained with 99% d.e. and e.e. after a single crystallization.

Influenza viruses pose a serious threat to world public health. Two of the drugs currently used to treat influenza patients are (−)-oseltamivir phosphate (Tamiflu) [140] and zanamivir (Relenza) [141]. Enamine catalysed transformations were exploited in the realisation of synthetic sequences leading to (−)-oseltamivir. The phosphate salt of this molecule, commercialised as Tamiflu, is a neuraminidase inhibitor useful as antiviral agent for the treatment and the prevention of influenza infections. Due to its specific biological action, (−)-oseltamivir is considered to be very general against all types of influenza viruses, including the avian H5N1, which has a mortality rate close to 50%. In the mid-2010s, fear for a potentially disastrous pandemic spread of a mutation of this virus amongst humans has prompted several nations to plan the accumulation of massive stocks of this drug. The production of this molecule is being based on a multistep synthesis employing shikimic acid as starting material. Both the variable availability of natural shikimic acid and the relative length of the synthesis put great pressure on industrialists and academics to develop very quickly alternative synthetic routes, in order to satisfy the exponentially increasing requests [142]. Asymmetric catalysis was considered a very attractive method, as it would not rely heavily on natural chiral sources. Accordingly, several syntheses based on enantioselective Lewis acid catalysis have been reported [143], starting already from 2006 [144, 145]. Enamine catalysis was later appreciated as a useful alternative and was consequently applied as the key stereodetermining step in a few syntheses of (−)-oseltamivir. Interestingly, many of the reported protocols went beyond the study of the simple reaction steps but increased the overall efficiency by combining many of the steps in a one pot fashion.

**Scheme 31:**
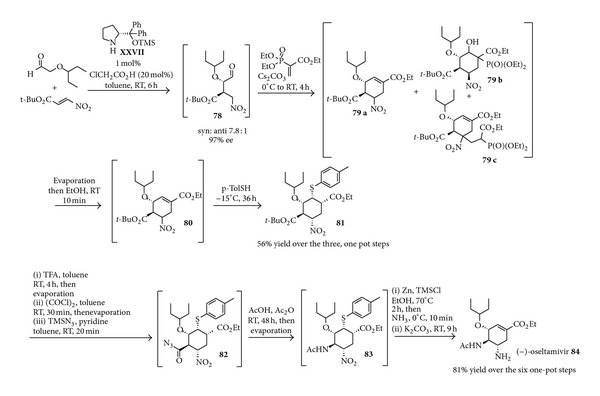
Two-pot organocatalysed synthesis of (−)-oseltamivir** 84**.

Essentially, two complementary organocatalytic strategies to (−)-oseltamivir have been developed, both based on the addition of an *α*-alkoxy aldehyde (pentan-3-yloxyacetaldehyde) to a nitroalkene. The first approach, disclosed in 2009 [146], involved the addition of this aldehyde to a 2-ester substituted nitroalkene, catalysed by a silyl-protected D-prolinol derivative. This constituted the first reaction of a nine-step synthesis leading to the target compound (−)-oseltamivir, in which all steps were carefully optimised and designed for their combination in one-pot sequences. The whole sequence could be in fact carried out in only three one-pot operations, which soon after [147] were reduced to two in the second generation synthesis depicted in Scheme 31. The synthesis starts with the mentioned organocatalytic reaction. The nitroaldehyde product** 78**, generated with high enantioselectivity, reacts then with a vinyl phosphonate in the presence of a base, giving a nitro-Michael Horner-Wadsworth-Emmons sequential reaction which however leads to a mixture of different products** 79 a**–**c**. Solvent evaporation followed by stirring the basic mixture in ethanol triggers retroaldol elimination processes followed by olefination rendering very nicely a single cyclohexene product** 80**, which upon treatment with a thiophenol gives a cyclic five-substituted cyclohexane** 81** featuring the desired stereochemistry, isolated in overall 56% yield over the three steps. The introduction of the thiophenol is necessary to control the relative configuration at the *α*-nitro chiral centre through equilibration. This intermediate (**81**) is converted into the target (−)-oseltamivir** 84** through a six-step one-pot sequence, starting with* tert*-butyl ester cleavage with trifluoroacetic acid, leading to an acid which is converted first in a chloride and then in an acylazide** 82**.

This azide undergoes a Curtius rearrangement with concomitant acetylation, which serves to install the desired acetamide functionality (**83**). Nitro group reduction with zinc, ammonia bubbling, and elimination of the stereodirecting thiophenol through a retro-Michael addition finally leads to the target oseltamivir product** 84**. It is worth noting that the metal-based reagents employed in this synthesis contain either alkali-metal ions or nontoxic Zn. Thus this procedure is suitable for large-scale preparation.

**Scheme 32:**
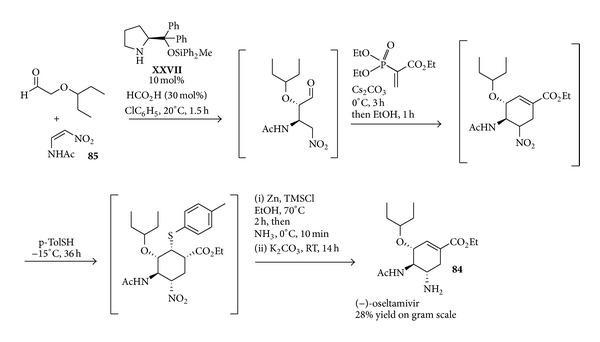
Scalable organocatalysed short synthesis of (−)-oseltamivir** 84**.

A second approach to this target molecule through organocatalysis aimed at avoiding the hazardous azide addition and Curtius rearrangement steps, by introducing the amide functionality already at the beginning of the synthesis. To this end, the readily available 2-Z-acetamido nitroethene** 85** was engaged in an enamine catalysed reaction with the same aldehyde previously employed. By this approach, disclosed in 2010 [148], the target oseltamivir** 84** could be obtained after a few more steps based on the previous synthesis. Due to its importance, this organocatalytic reaction and the subsequent steps were then the subject of thorough studies directed at their generalisation [149, 150] and careful optimisation [151], which culminated very recently in the disclosure of a synthesis of oseltamivir proceeding in one-pot from the aldehyde and this nitroalkene [152]. Compared to the previous sequence reported in Scheme 31, this synthesis (Scheme 32) not only obviates the need of introducing the acetamide functionality with azide chemistry, but also avoids evaporation steps and the usage of toxic chlorinated solvents, allowing the obtainment of oseltamivir even on gram scale with a remarkable 28% yield over the five steps without any intermediate work-up or isolation.

In the same year a nine-step synthesis of (−)-oseltamivir has been proposed [153] in which a novel barium-catalysed asymmetric Diels-Alder-type reaction gives access to the cyclohexene framework of  Tamiflu in the key enantioselective reaction path with a Pd-catalysed allylic occurring in the following steps of the synthetic sequence. Though of relevant interest for employing a nontoxic metal and for being effective at 60 g scale, the advantage of this synthesis with respect to the organocatalysed methodology is to some extent diminished by the need of isolating all the intermediates and by the use of a potentially explosive reagent such as diphenyl phosphoryl azide.

## 3. Concluding Remarks

Featuring aspects of medicinal chemistry are the strong reliability of the processes, the use of cheap and commercial catalysts, and short synthetic sequences. Moreover, both enantiomers of the catalysts should be easily available in order to obtain both enantiomers of the products and allow analysing them from a biological viewpoint. As herein highlighted, enantioselective organocatalysis plays nowadays a major role, alongside metal-catalysed processes, in chemical synthesis because together these complementary disciplines have revolutionized the way the synthesis is carried out. A point that should not be underestimated is the attractiveness of the operational simplicity of organocatalytic reactions: rigorous exclusion of oxygen and moisture is usually not required, and potential toxic metal contamination is avoided. The success and the usefulness of the organocatalytic approach are well demonstrated by the several examples in which different organocatalytic strategies, involving various activation modes and/or catalyst structures, have been successfully applied to the same target compound, as herein exemplified in the case of baclofen or rolipram.

The application of organocatalysis to the synthesis of molecules of interest in various fields including medicinal chemistry is however still facing problems such as the relatively high catalyst loading, the long reaction time, and also the difficult recyclability of the organocatalyst. To give a partial solution, significant advances have been reported in recent times describing polymer supported organic catalysts, easily recovered after the reaction has taken place by filtration [154–156]. In contrast with immobilised metal complexes (via solid-supported bound ligand), leaching problems are much less critical when using organocatalysts immobilised by covalent bonding to the solid support. Even if costs considerations, as well as decrease of catalyst activity and selectivity, are issues not fully solved yet, several remarkable examples have been reported, which show great promise for future development.

## Figures and Tables

**Table 1:** Catalytic asymmetric approaches to warfarin through iminium ion catalysis.

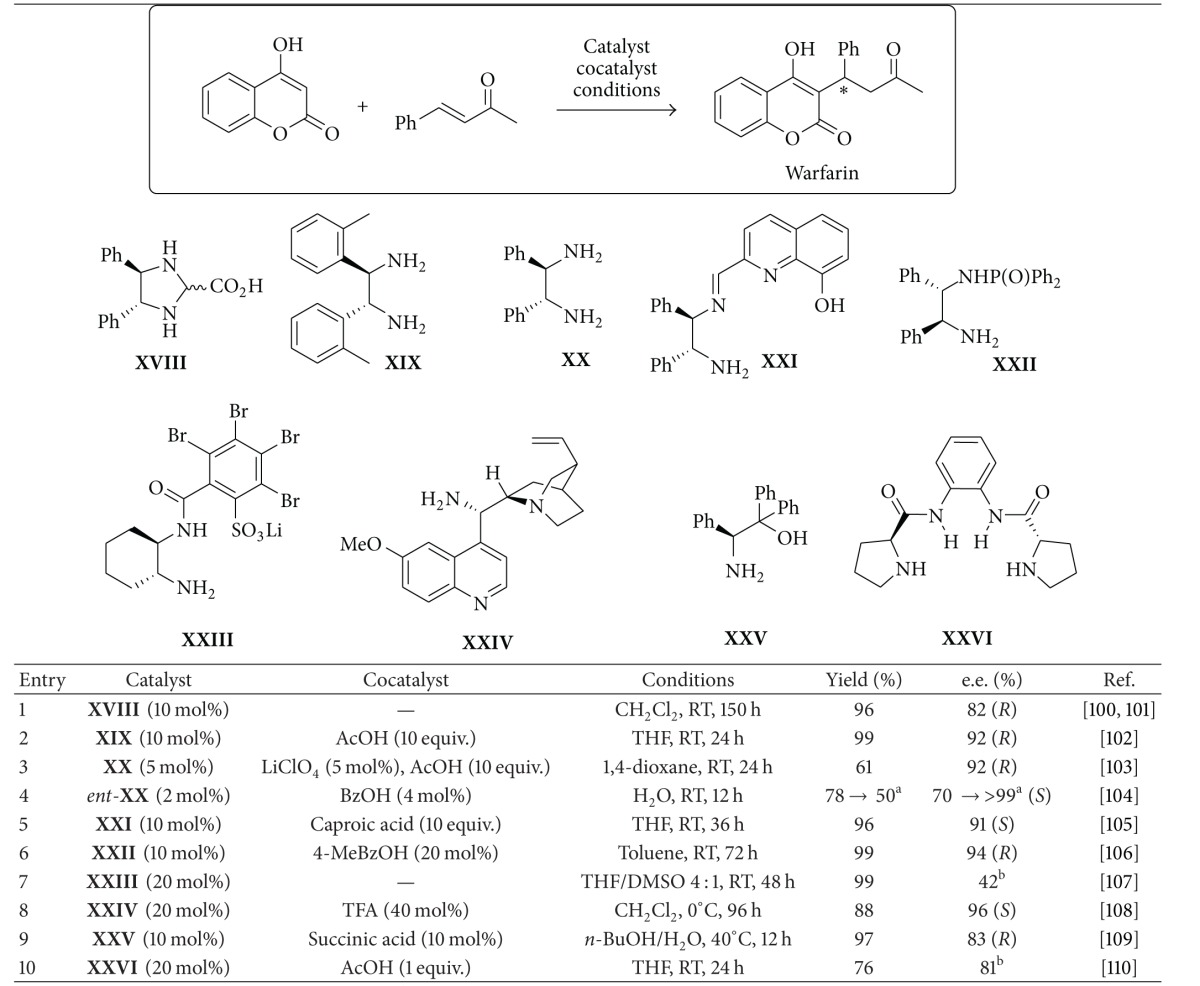

^a^Isolated by precipitation from the reaction mixture. ^b^Absolute configuration not reported.
